# A Novel Scale for Assessment of Stroke Severity at Symptom Onset: Correlation With Neurological Deterioration and Outcome

**DOI:** 10.3389/fneur.2020.602839

**Published:** 2021-01-22

**Authors:** Qi Li, Lan Deng, Cheng Huang, Wen-Yu Zhang, Ning Zou, Du Cao, Xiao Wei, Xin-Yue Qin

**Affiliations:** ^1^Department of Neurology, The First Affiliated Hospital of Chongqing Medical University, Chongqing, China; ^2^Department of Neurology, Panzhihua Municipal Central Hospital, Panzhihua, China; ^3^Department of Traditional Chinese Medicine, Chongqing Medical and Pharmaceutical College, Chongqing, China

**Keywords:** acute ischemic stroke, scale, prehospital stroke scale, ultraearly neurological deterioration, functional outcome

## Abstract

**Objective:** To propose a novel scale for the assessment of stroke severity at symptom onset and to investigate whether it is associated with ultra-early neurological deterioration (UND) and functional outcomes.

**Methods:** The Chongqing Stroke Scale (CQSS) was constructed based on key aspects of history, emphasizing language, motor function, and level of consciousness to yield a total 0–11 scale. The diagnostic performance of the CQSS was assessed in 215 ischemic stroke patients between June 2017 and October 2017 in a tertiary hospital. Patients were included if they presented within 24 h after onset of symptoms and they or their witness can recall the scenario at symptom onset. UND was defined as an increase ≥2 points on the CQSS between symptom onset and admission. Functional outcomes were assessed using the 3-month modified Rankin scale. The correlation between the CQSS score and baseline National Institutes of Health Stroke Scale (NIHSS) score was assessed. The sensitivity, specificity, and positive and negative predictive values of CQSS for the outcomes were calculated. Logistic regression was used to test the association between the CQSS score and functional outcomes.

**Results:** A total of 215 patients with available CQSS scores were included. Baseline CQSS scores at symptom onset were correlated with the admission NIHSS score (*r* = 0.56, *p* < 0.001) and functional outcome at 3 months (*r* = 0.47, *p* < 0.001). Baseline CQSS ≥ 6 was an independent predictor of functional outcome at 3 months (odds ratio, 12.61; 95% confidence interval 5.68–27.97, *p* < 0.001). UND was observed in 20 (9.30%) patients. The 90-day mortality was significantly higher in patients with UND than those without UND (25.0 *vs*. 8.2%, *p* < 0.001). After adjusting for age, admission systolic blood pressure, hypertension, and diabetes, UND independently predicted poor functional outcome in the multivariate logistic regression model (odds ratio, 9.69; 95% confidence interval 3.19–29.45, *p* < 0.001).

**Conclusions:** The newly developed CQSS is a simple and easy-to-perform scale that allows a quantitative evaluation of the stroke severity at symptom onset and an assessment of UND before hospital admission. It is associated with NIHSS and predicts functional outcome in patients with acute ischemic stroke.

## Introduction

Acute ischemic stroke is the second leading cause of death and morbidity worldwide (1). A reliable measurement of neurological deficits after the event is important for the triage of patients for specific treatment and prognostic stratification. Several clinical grading scales have been developed and widely used in clinical practice to assess stroke severity. The National Institutes of Health Stroke Scale (NIHSS) has been proven to be useful in the routine assessment of neurological deficit in patients with stroke and has been widely used in the context of clinical trials (2). Recently, a shortened version of the NIHSS has been developed for the rapid assessment of neurological deficits in a timely manner (3–6). However, the NIHSS remains a complex neurological scale that requires professional training for an accurate assessment. Prehospital assessment of neurological status is also important for the rapid identification and management of potential stroke patients. Several prehospital scales such as the Los Angeles Motor Scale, shortened NIHSS for emergency medical services, the Los Angeles Prehospital Stroke Screen, and Cincinnati Prehospital Stroke Scale have been developed for rapid identification of stroke in emergency settings (4, 5). These tools are useful diagnostic instruments that permit emergency medical staff to identify and triage stroke patients with high sensitivity and specificity.

Current stroke clinical identification and assessment scales, such as the NIHSS, the European Stroke Scale, Los Angeles Prehospital Stroke Screen, and Cincinnati Prehospital Stroke Scale, are designed to identify stroke and describe the severity of neurologic deficits in patients already diagnosed with stroke (7–9). All existing scales do not provide any information regarding the stroke severity at the onset. However, documentation of the severity and type of symptoms at stroke onset is important for understanding the natural history of stroke.

The earliest time point from which the neurological status can be recorded is the emergency service assessment or at hospital admission. Previous studies have reported early neurological deterioration (END), which was defined as worsening of clinical symptoms assessed by an increase in NIHSS score of 2 or 4 points within 48 h of admission (10–12). The neurological deterioration has been frequently observed in patients with stroke and has been associated with worse functional outcomes (11, 12). Recent studies have suggested that neurological deterioration may occur during the prehospital period (13). However, the change in neurological function between symptom onset and the first clinical evaluation, i.e., emergency service assessment or at hospital admission, has not been assessed. The neurological deterioration usually occurs immediately after symptom onset, and there is an urgent need to understand the natural history of stroke evolution between symptom onset and clinical assessment. We proposed the term ultra-early neurological deterioration (UND) to identify patients who had clinical worsening between symptom onset and the first clinical assessment.

We designed a new stroke diagnostic scale, the Chongqing Stroke Onset Scale (CQSS) to document the severity of stroke at symptom onset. The study aimed to evaluate the association between the severity of stroke symptoms at onset and admission stroke severity and functional outcomes in patients with acute ischemic stroke.

## Methods

The CQSS was designed by Dr. Qi Li based on the clinical history of patients with stroke. The scale focuses on key aspects of history, emphasizing language, motor function, and level of consciousness. Table 1 displays the scoring sheet of the stroke scale. The CQSS evaluates the language, motor function, and level of consciousness. Each item on the scale that evaluates the language and motor function of the stroke scale was graded 0–3, where 0 indicated normal, one mild, two severe abnormalities, and three total loss of function. The level of consciousness was graded 0–2, where zero indicated normal, one drowsy, and two coma. This stroke scoring system yields a deficit score that ranges from 0 (normal) to 11 (most affected) in patients with stroke.

**Table 1:** Chongqing stroke scale.

Language	□ 0 = Normal; no noticeable language impairment □ 1 = Mild-to-moderate language impairment; some loss of fluency or slurred speech, without significant limitation on expression. Patient's speech can be understood □ 2 = Severe language impairment; fragmentary expression or severely slurred speech, patient's speech is unintelligible □ 3 = Mute or produce a single word or unintelligible sound, no usable speech
Motor Upper Limb	□ 0 = Normal; no noticeable upper limb weakness □ 1 = Mild-to-moderate impairment; patient feels mild weakness in the upper limb and is still able to hold daily objects or raise arms □ 2 = Severe impairment; patient feels severe weakness in the upper limb and cannot hold daily objects or raise arms but makes movements □ 3 = No movement
Motor Lower Limb	□ 0 = Normal; no noticeable lower limb weakness □ 1 = Mild-to-moderate impairment; patient feels mild weakness in the lower limb and is still able to stand unaided □ 2 = Severe impairment; patient feels severe weakness in the lower limb and cannot stand unaided, but makes movements □ 3 = No movement
Level of Consciousness	□ 0 = Alert □ 1 = Drowsy, decreased level of consciousness but can respond to verbal or painful stimuli □ 2 = Coma, unresponsive

Consecutive stroke patients at the First Affiliated Hospital of Chongqing Medical University who presented with stroke between June 2017 and October 2017 were prospectively evaluated with our newly derived stroke scale. The patient or the witness was asked to recall the scenario at the onset. The admission stroke severity was measured by the NIHSS, with higher scores indicating a more severe neurological deficit. The functional outcome was assessed using the modified Rankin scale (mRS) at 3 months. Functional independence was defined as an mRS score of 0–2 (2, 3).

The performance of the CQSS was assessed, and patients were included in the final analysis if they or their witnesses could recall the scenario at symptom onset. Patients who presented after 24 h were excluded. Patients with wake-up stroke or stroke of unknown onset were also excluded from the study. The CQSS was assessed for symptom onset, and the admission CQSS was derived from the arm and leg strength, language, and level of consciousness items of the NIHSS. UND was defined as an increase ≥2 points on the CQSS between admission and symptom onset in patients who presented within 24 h after the ictus. The prevalence, clinical characteristics, and association between UND and functional outcome were assessed.

### Statistical Analyses

All statistical analyses were performed using SPSS 23.0 software. Continuous variables are reported as the mean ± standard deviation (SD) or as the median ± interquartile range (IQR) as appropriate. All categorical variables were reported as proportions. The linear correlation of the CQSS and the NIHSS scores was analyzed using the Spearman test. The sensitivity, specificity, positive predictive value, negative predictive value, and accuracy were assessed. The optimal thresholds of the CQSS and UND were evaluated using overall accuracy. The interclass correlation coefficient was used to assess interobserver variability. Spearman's correlation coefficient was used to test the association between baseline CQSS and baseline NIHSS score and functional outcome. The distribution of the CQSS was compared. Logistic regression was performed to assess the predictive ability of CQSS (≤ 6 *vs*. >6) in terms of functional outcome. A *P*-value of < 0.05 was considered statistically significant.

### Standard Protocol Approvals, Registrations, and Patient Consents

The First Affiliated Hospital of Chongqing Medical University Institutional Review Board approved this study. Written informed consent was obtained from each participant or their legal representatives.

## Results

A total of 567 consecutive patients presented with acute ischemic stroke between June 2017 and October 2017. After applying the exclusion criteria, we excluded 240 patients who arrived after 24 h and 97 patients with wake-up stroke or stroke of unknown onset. A total of 10 patients who were lost to follow-up at 3 months were also excluded. The CQSS scale was not evaluated in five patients who were unable to communicate, and the witnesses were not approached. A total of 215 patients [mean age 68.1 years (SD 12.6); 79 women and 136 men] were included in the final analysis.

Among the 215 patients with available CQSS scores, 79 (36.8%) had scores of 0–2, 91 (42.3%) had scores of 3–5, 34 (15.8%) had scores of 6–8, and 11 (5.1%) had scores above 8. Fourteen patients had a pre-stroke mRS score above two at admission. The average NIHSS score of patients with CQSS > 5 was 5.9 (range 0–34), and the average for patients with CQSS <6 was 6.0 (range 0–34). In total, 149 (69.3%) of these 215 patients were independent, 45 (20.9%) were dependent, and 21 (9.8%) had died at 3 months. Patients with poor functional outcomes were older and more likely to have higher baseline NIHSS scores, lower Glasgow Coma Scale (GCS) scores, and higher CQSS scores at symptom onset. The baseline demographic, clinical, and radiological characteristics between patients with and without poor outcomes (mRS 3–6) are illustrated in Table 2.

**Table 2:** Comparison of baseline demographic, clinical, and radiological characteristics between patients with and without poor outcome (mRS 3–6).

**Variables**	**Poor Outcome (*n* = 66, 30.7%)**	**Favorable Outcome (*n* = 149, 69.3%)**	***P*-Value**
**Demographic**			
Mean age, year (SD)	70.2 (14.7)	67.2 (11.5)	0.154
Sex, male, *n* (%)	40 (60.6)	96 (64.4)	0.592
**Medical history**			
Alcohol consumption, *n* (%)	14 (21.2)	39 (26.2)	0.436
Smoking, *n* (%)	30 (45.5)	71 (47.7)	0.766
Coronary disease, *n* (%)	22 (33.3)	28 (18.8)	0.020
Hypertension, *n* (%)	38 (57.6)	88 (59.1)	0.838
Diabetes mellitus, *n* (%)	18 (27.3)	40 (26.8)	0.948
**Clinical features**			
Systolic blood pressure, mmHg (SD)	155.9 (29.6)	149.4 (21.6)	0.109
Diastolic blood pressure, mmHg (SD)	84.9 (15.9)	85.7 (14.4)	0.713
Admission GCS score, median (IQR)	14.0 [13.0–15.0]	15.0 [15.0–15.0]	<0.001
Admission NIHSS score, median (IQR)	9.5 [5.0–17.0]	2.0 [1.0–3.0]	<0.001
CQSS score, (IQR)	5.5 [3.8–7.0]	3.0 [1.5–4.0]	<0.001
Time to admission (IQR)	6.5 [2.5–13.1]	5.0 [3.0–11.3]	0.927

*GCS, Glasgow Coma Scale; NIHSS, National Institutes of Health Stroke Scale; IQR, interquartile range; SD, standard deviation; mRS, modified Rankin Scale; CQSS, Chongqing Stroke Onset Scale*.

The interclass correlation coefficient of the CQSS was high, with a value of 0.88 (*p* < 0.001, 95% confidence interval 0.66–0.94). The interclass median baseline CQSS value at symptom onset was 3.0 (IQR 2.0–5.0), and the median CQSS at hospital admission was 2.0 (IQR 1.0–5.0). The baseline NIHSS score was 4.0 (IQR 1.0–7.0). As expected, there was a strong correlation between admission CQSS and admission NIHSS score (rs = 0.91, *p* < 0.001).

We observed that the CQSS at symptom onset correlated with the admission NIHSS score (rs = 0.56, *p* < 0.001) and functional outcome at 3 months (rs = 0.47, *p* < 0.001). A CQSS of <6 at symptom onset discriminated functional independence and death at 3 months. The optimal cutoff value of the CQSS for predicting poor functional outcome was assessed; it is illustrated in Table 3. The best accuracy was achieved for a score of 6, with a sensitivity and specificity of 50.0 and 91.9%, respectively, for predicting poor outcome. Patients with CQSS ≥ 6 had higher NIHSS scores (13.0 vs. 3.0; *p* < 0.001, Table 4).

**Table 3:** Sensitivity, specificity, positive predictive value, and negative predictive value of different cutoff values of the CQSS score for predicting poor outcome.

**CQSS score**	**sensitivity**	**specificity**	**PPV**	**NPV**	**OA**
≥1 (*n* = 200)	1.000	0.101	0.330	1.000	0.377
≥2 (*n* = 173)	0.924	0.248	0.353	0.881	0.456
≥3 (*n* = 136)	0.803	0.443	0.390	0.835	0.553
≥4 (*n* = 95)	0.758	0.698	0.526	0.867	0.716
≥5 (*n* = 72)	0.682	0.819	0.625	0.853	0.777
≥6 (*n* = 45)	0.500	0.919	0.733	0.806	0.791
≥7 (*n* = 32)	0.348	0.940	0.719	0.765	0.758
≥8 (*n* = 13)	0.182	0.993	0.923	0.733	0.744
≥9 (*n* = 11)	0.152	0.993	0.909	0.725	0.735
≥10 (*n* = 4)	0.061	1.000	1.000	0.706	0.712
11 (*n* = 3)	0.045	1.000	1.000	0.703	0.707

*Overall accuracy (OA) represents true positive plus true negative divided by total number of patients (n = 215).

*CQSS, Chongqing Stroke Onset Scale; PPV, positive predictive value; NPV, negative predictive value; OA, overall accuracy*.

**Table 4:** Comparison of baseline demographic, clinical, and radiological characteristics between patients with CQSS score ≥6 and those without.

**Variables**	**CQSS score ≥ 6 (*n* = 45)**	**CQSS score <6 (*n* = 170)**	***P*-Value**
**Demographic**			
Mean age, year (SD)	69.6 (15.6)	67.7 (11.7)	0.456
Sex, male, *n* (%)	21 (46.7)	115 (67.6)	0.009
**Medical history**			
Alcohol consumption, *n* (%)	9 (20.0)	44 (25.9)	0.416
Smoking, *n* (%)	14 (31.1)	87 (51.2)	0.016
Hypertension, *n* (%)	29 (64.4)	97 (57.1)	0.371
Diabetes mellitus, *n* (%)	15 (33.3)	43 (25.3)	0.280
Coronary disease, *n* (%)	17 (37.8)	33 (19.4)	0.010
**Clinical features**			
Systolic blood pressure, mmHg (SD)	153.4 (26.6)	150.9 (23.9)	0.544
Diastolic blood pressure, mmHg (SD)	83.8 (13.9)	85.9 (15.0)	0.416
Admission GCS score, median (IQR)	14.0 [13.0–15.0]	15.0 [15.0–15.0]	<0.001
Admission NIHSS score, median (IQR)	13.0 [7.0–17.0]	3.0 [1.0–5.0]	<0.001
Time to admission, median (IQR)	4.0 [2.5–10.3]	6.0 [3.0–13.5]	0.223
pre-hospital care and aid, *n* (%)	28 (62.2)	46 (27.2)	<0.001
thrombolysis / thrombectomy, *n* (%)	11 (24.4)	23 (13.6)	0.077
**Outcome**			
90-day mRS score, median (IQR)	3.0 [2.0–6.0]	1.0 [0–2.0]	<0.001
90-day mRS 3–6, *n* (%)	33 (73.3)	33 (19.4)	<0.001

*GCS, Glasgow Coma Scale; NIHSS, National Institutes of Health Stroke Scale; IQR, interquartile range; SD, standard deviation; mRS, modified Rankin Scale; CQSS, Chongqing Stroke Onset Scale*.

After controlling for age, hypertension, diabetes, and baseline admission systolic blood pressure, baseline CQSS ≥ 6 was an independent predictor of functional outcome at 3 months (odds ratio, 12.61; 95% confidence interval 5.68–27.97, *p* < 0.001). The baseline CQSS value was correlated with the severity of stroke on the NIHSS on admission (rs = 0.56, *p* < 0.001). The baseline CQSS value predicted functional outcome and early neurological deterioration.

### Ultra-Early Neurological Deterioration

The best overall accuracy of UND for predicting poor outcome was achieved for a score of ≥2 (Table 5). The sensitivity, specificity, and positive and negative predictive values of UND for predicting poor outcome were 21.2, 96.0, 70.0, and 73.3%, respectively.

**Table 5:** Prognostic value of different cutoff values of the CQSS score increase for the poor outcome.

**CQSS score increase**	**sensitivity**	**specificity**	**PPV**	**NPV**	**OA**
≥1	0.424	0.819	0.509	0.763	0.698
≥2	0.212	0.960	0.700	0.733	0.730
≥3	0.136	0.980	0.750	0.719	0.721
≥4	0.091	0.993	0.857	0.712	0.716
≥5	0.061	1.000	1.000	0.706	0.712

*Overall accuracy (OA) represents true positive plus true negative divided by total number of patients (n = 215).

*CQSS, Chongqing Stroke Onset Scale; PPV, positive predictive value; NPV, negative predictive value; OA, overall accuracy*.

**Figure 1:**
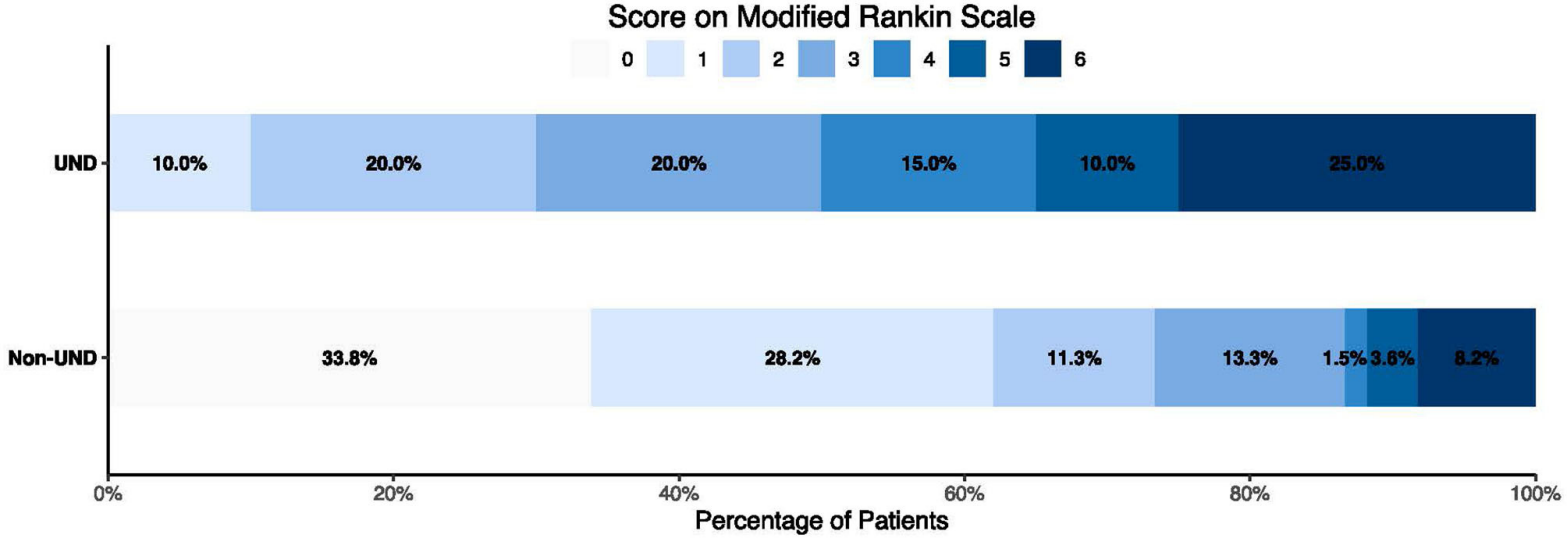
Distribution of modified Rankin Scale score in patients with or without ultra-early neurological deterioration.

A total of 20 patients (9.3%) had UND, defined as an increase of more than one point on the CQSS between symptom onset and admission. Of those, 14 (70.0%) had poor functional outcomes. The 90-day mortality was significantly higher in patients with UND than in those without (25.0 *vs*. 8.2%, *P* < 0.001, Table 6). The distribution of the 3-month mRS score in patients with UND and those without is illustrated in Figure 1. After adjusting for age, admission systolic blood pressure, hypertension, and diabetes, UND independently predicted poor functional outcome in the multivariate logistic regression model (odds ratio, 9.69; 95% confidence interval 3.19–29.45, *p* < 0.001).

**Table 6:** Comparison of baseline demographic, clinical, and radiological characteristics between patients with ultra-early neurological deterioration and those without.

**Variables**	**UND (*n* = 20)**	**Non-UND (*n* = 195)**	***P*-Value**
**Demographic**			
Mean age, year (SD)	63.0 (14.9)	68.7 (12.3)	0.056
Sex, male, *n* (%)	12 (60.0)	124 (63.6)	0.751
**Medical history**			
Alcohol consumption, *n* (%)	9 (45.0)	44 (22.6)	0.027
Smoking, *n* (%)	12 (60.0)	89 (45.6)	0.220
Hypertension, *n* (%)	10 (50.0)	116 (59.6)	0.412
Diabetes mellitus, *n* (%)	5 (25.0)	53 (27.2)	0.834
Coronary disease, *n* (%)	4 (20.0)	46 (23.6)	0.717
**Clinical features**			
Systolic blood pressure, mmHg (SD)	144.7 (35.1)	152.1 (23.1)	0.364
Diastolic blood pressure, mmHg (SD)	83.8 (17.3)	85.6 (14.6)	0.608
Admission GCS score, median (IQR)	13.5 [12.0–15.0]	15.0 [15.0–15.0]	<0.001
Admission NIHSS score, median (IQR)	12.0 [7.5–18.5]	3.0 [1.0–7.0]	<0.001
Time to admission, median (IQR)	6.3 [2.4–12.4]	5.0 [3.0–13.0]	0.176
pre-hospital care to aid, *n* (%)	14 (70.0)	60 (30.9)	<0.001
thrombolysis/thrombectomy, *n* (%)	5 (15.9)	29 (14.9)	0.242
**Outcome**			
90-day mRS score, median (IQR)	3.5 [2.0–5.8]	1.0 [0–3.0]	<0.001
90-day mRS 3–6, *n* (%)	14 (70.0)	52 (26.7)	<0.001

*GCS, Glasgow Coma Scale; NIHSS, National Institutes of Health Stroke Scale; IQR, interquartile range; SD, standard deviation; mRS, modified Rankin scale; CQSS, Chongqing Stroke Onset Scale; UND, ultra-early neurological deterioration*.

## Discussion

In our study, we have designed a practical scale that allows us to assess the severity of symptom onset in patients with stroke. To the best of our knowledge, this is the first stroke scale that allowed us to document the severity of stroke at symptom onset. Our results demonstrated that CQSS correlated with the admission NIHSS and functional outcome at 3 months after stroke. More importantly, we observed that 9.3% of patients with acute ischemic stroke might experience UND before hospital admission. We demonstrated that patients with UND are associated with worse functional outcomes.

Existing stroke scales, such as the NIHSS, Scandinavian Stroke Scale, and the Canadian Neurological Scale, are designed to assess the severity of neurologic deficits in patients with stroke (2, 3, 14–18). The NIHSS is the most widely used clinical tool for the assessment of stroke severity, and the scale correlates well with different measures of functional outcome. Adequate training is required for accurate assessment of neurological deficits (19). The Los Angeles Motor Scale allows rapid characterization of stroke severity in prehospital settings and demonstrates a correlation with the NIHSS and 3-month functional outcome (3). More recently, Purrucker et al. devised a combined stroke recognition, severity scale, and large vessel occlusion detection instrument (shortened NIHSS for emergency medical services) that allows for evaluation of stroke progression starting at the prehospital phase (6). However, these scales require professional skills, and the neurological deficit at symptom onset could not be assessed by any existing stroke scale. The CQSS is a simple and easy-to-use scale that can be performed without a detailed physical examination of the patients. Unlike existing stroke scales, the CQSS is based on a detailed document of the patient history. It offers an opportunity to document the true natural history of stroke evolution. The CQSS is a simple stroke scale that consists of only three items (language, motor function, and level of consciousness). In our study, language, motor function, and level of consciousness were assessed because they are easy to obtain based on detailed inquiries about the history. More importantly, the language and motor function are the most robust features of almost all existing stroke scales and prehospital stroke scales. Although the NIHSS is the most widely used and robust method for measuring neurological deficits in patients with stroke, it requires a detailed examination of patients by rigorously trained neurologists. Thus, it is hard for primary care doctors or paramedical staff to perform this scale. Unlike the NIHSS, the CQSS does not require professional skills in evaluating the deficits and could be more widely used in prehospital and community settings where medical resources are limited.

In our study, we demonstrated that the stroke severity as assessed by the CQSS at symptom onset correlated well with the admission NIHSS score (rs = 0.56, *p* < 0.001). More importantly, the admission CQSS was strongly correlated (correlation coefficient of 0.88, *p* < 0.001) with the admission NIHSS score, which is the gold standard measurement of neurological deficits in patients with stroke. The highest overall accuracy (79.1%) was achieved for a score of 6, with a positive predictive value of 73.3% and a negative predictive value of 80.6% for predicting poor outcome.

The neurological deficit at symptom onset is an important component of the natural history of stroke, and understanding of stroke evolution before admission to the hospital is important for triage and prognostic stratification of patients. The CQSS may be used as a tool for objective assessment of symptom change between the onset of symptoms and hospital admission. The early neurological deterioration reflects changes in neurological deficit after hospital admission. However, the established criteria only reflect the change of neurological status after admission. Our newly developed CQSS provides a useful tool to assess the neurological status changes before admission.

In our study, we proposed a new concept of UND to describe UND between symptom onset and admission. To our knowledge, this is the first investigation of neurological deterioration immediately after symptom onset in patients with acute ischemic stroke. In previous studies, neurological deterioration has been assessed solely in patients who were admitted to the hospital or at the emergency medical service. The reported rate of END after admission ranges from 4 to 20% and depends on the definitions (11, 20, 21). Early neurological deterioration was defined as an increase in NIHSS score of ≥2 or ≥4 points and has been consistently associated with poor outcomes (10–12). In our study, we found that a significant proportion of patients (9.3%) may experience neurological deterioration immediately after symptom onset and before admission to the hospital. In these patients, 70% will have a worse functional outcome. Because the ultimate goal of treatment is to prevent END and improve functional outcomes, timely identification of patients with UND is important for patient triage and management.

Our study has several limitations. First, our newly developed scale relies on the detailed history taken from the patient or the witness who knows the scenario at symptom onset. Therefore, the CQSS could not be performed to assess the initial neurological deficit in patients with stroke of unknown onset. Second, some patients with atypical stroke symptoms could be missed based on the stroke scale. Third, this is a single-center study, and external validation with different cohorts is needed. Forth, patients' or witnesses' recall of symptoms might vary, and the accuracy of the scale cannot be demonstrated, as there is no reference standard. The disagreement between onset and admission may be due to the non-expert and expert CQSS assessment at different time points. It may be difficult to generalize to other clinical contexts where prehospital transport times may differ. Fifth, the UND group was small, and variability in scoring might account for the association.

In conclusion, the newly developed CQSS is a simple and easy-to-perform scale that documents the severity of neurological deficits at symptom onset. It does not require neurological examination skills and would be easily performed in all clinical settings. The CQSS correlated with the admission NIHSS and independently predicted poor functional outcome at 3 months. Future studies with greater numbers of patients are needed to test whether the newly developed scale is reproducible in an independent population of patients with stroke.

## Data Availability Statement

The raw data supporting the conclusions of this article will be made available by the authors, without undue reservation.

## Ethics Statement

The studies involving human participants were reviewed and approved by the First Affiliated Hospital of Chongqing Medical University Institutional Review Board. The patients/participants provided their written informed consent to participate in this study.

## Author Contributions

QL: study concept and design. QL and LD: drafting the manuscript. QL, LD, CH, XW, and X-YQ: critical revision of the manuscript for important intellectual content. QL, LD, CH, W-YZ, NZ, and DC: data collection. LD and XW: statistical analysis. QL: obtain funding. X-YQ: administrative support. All authors contributed to the article and approved the submitted version.

## Conflict of Interest

The authors declare that the research was conducted in the absence of any commercial or financial relationships that could be construed as a potential conflict of interest.
